# A brief history of nearly EV‐erything – The rise and rise of extracellular vesicles

**DOI:** 10.1002/jev2.12144

**Published:** 2021-12-17

**Authors:** Yvonne Couch, Edit I. Buzàs, Dolores Di Vizio, Yong Song Gho, Paul Harrison, Andrew F. Hill, Jan Lötvall, Graça Raposo, Philip D. Stahl, Clotilde Théry, Kenneth W. Witwer, David R. F. Carter

**Affiliations:** ^1^ Acute Stroke Programme, Radcliffe Department of Medicine University of Oxford, John Radcliffe Hospital, Headley Way, Headington Oxford UK; ^2^ Department of Genetics, Cell‐ and Immunobiology Semmelweis University Budapest Hungary; ^3^ ELKH‐SE Immune‐Proteogenomics Extracellular Vesicle Research Group Budapest Hungary; ^4^ HCEMM‐SU Extracellular Vesicles Research Group Budapest Hungary; ^5^ Department of Surgery Pathology & Laboratory Medicine Cedars‐Sinai Medical Center Los Angeles California USA; ^6^ Department of Life Sciences Pohang University of Science and Technology Pohang Republic of Korea; ^7^ Institute of Inflammation and Ageing College of Medical and Dental Sciences University of Birmingham Edgbaston Birmingham UK; ^8^ Department of Biochemistry and Genetics La Trobe Institute for Molecular Science La Trobe University Bundoora Victoria Australia; ^9^ Krefting Research Centre Institute of Medicine Sahlgrenska Academy at University of Gothenburg Gothenburg Sweden; ^10^ Institut Curie Paris Sciences et Lettres Research University Centre National de la Recherche Scientifique UMR144, Structure and Membrane Compartments Paris France; ^11^ Department of Cell Biology Washington University School of Medicine St Louis Missouri USA; ^12^ INSERM U932 Institut Curie Paris Sciences et Lettres Research University Paris France; ^13^ Molecular and Comparative Pathobiology and Neurology, and The Richman Family Precision Medicine Center of Excellence in Alzheimer’s Disease The Johns Hopkins University School of Medicine Baltimore Maryland USA; ^14^ Department of Biological and Medical Sciences Faculty of Health and Life Sciences Oxford Brookes University Oxford UK; ^15^ Evox Therapeutics Limited Oxford Science Park Oxford OX4 4HG UK

**Keywords:** ectosome, exosome, extracellular vesicle, microparticle, microvesicle

## Abstract

Extracellular vesicles (EVs) are small cargo‐bearing vesicles released by cells into the extracellular space. The field of EVs has grown exponentially over the past two decades; this growth follows the realisation that EVs are not simply a waste disposal system as had originally been suggested by some, but also a complex cell‐to‐cell communication mechanism. Indeed, EVs have been shown to transfer functional cargo between cells and can influence several biological processes. These small biological particles are also deregulated in disease. As we approach the 75th anniversary of the first experiments in which EVs were unknowingly isolated, it seems right to take stock and look back on how the field started, and has since exploded into its current state. Here we review the early experiments, summarise key findings that have propelled the field, describe the growth of an organised EV community, discuss the current state of the field, and identify key challenges that need to be addressed.

## 1 THE EARLY EXPERIMENTS

The experiments in which EVs were specifically identified as biological entities, with enzymatic and functional potential, began during the 1980s and 1990s. Prior to this period there are numerous studies that hint at potential structures that *would subsequently* be described as EVs, or that describe experiments in which we can retrospectively speculate *may have* involved the activity of EVs. In this sense the story of the origins of EV research arguably begins with the studies of coagulation.

As a topic this dates back to the mid‐1600s and is covered in excellent reviews elsewhere (Hargett & Bauer, 2013; Quick, 1966). For the purposes of this article we will start with Chargaff and West and their studies on blood clotting, performed in New York in the 1940s. West was a clinician, with an on‐going interest in anaemia and haemophilia, and Chargaff was a biochemist. Chargaff had begun a series of papers in 1936 in the Journal of Biological Chemistry entitled *Studies on the Chemistry of Blood Coagulation* and made an observation in paper XIX of the series – *Cell Structure and the Problem of Blood Coagulation* – which can be interpreted as the beginning of the field of EV biology. When spinning down blood to establish a centrifugation protocol to separate clotting factors from cells, Chargaff observed that “*the addition of the high speed sediment to the supernatant plasma brought about a very considerable shortening of the clotting time*” (Chargaff, 1945). Enigmatically he went on to say “*this will be discussed in detail on a later occasion*”; that later occasion turned out to be his paper published with Randolph West in 1946 on *The Biological Significance of the Thromboplastic Protein of Blood*. Here they discovered a ‘particulate fraction’ which sedimented at 31,000 *g* and had high clotting potential, as well as a ‘thromboplastic protein’. The authors suggested that this fraction “*probably includes, in addition to the thromboplastic agent, a variety of minute breakdown products of blood corpuscles*” (Chargaff & West, 1946). However, it would be some years before these were specifically identified as EVs.

In fact, 17 years would pass until Peter Wolf described a “*material in minute particulate form, sedimentable by high‐speed centrifugation and originating from platelets, but distinguishable from intact platelets*” which we now know as the EV fraction. Wolf published electron microscopy images of these particles, which he described as ‘platelet dust’ (Wolf, 1967). Following this, in 1971, Neville Crawford published further images of these vesicles—which were now being described as ‘microparticles’—obtained from platelet‐free plasma. Crawford also showed they contained lipid and carried cargo including ATP and contractile proteins (Crawford, 1971). These pioneering experiments with platelets were the first to describe the presence and coarse structure of such cell‐free components and hinted at their potential biological importance.

**FIGURE 1:**
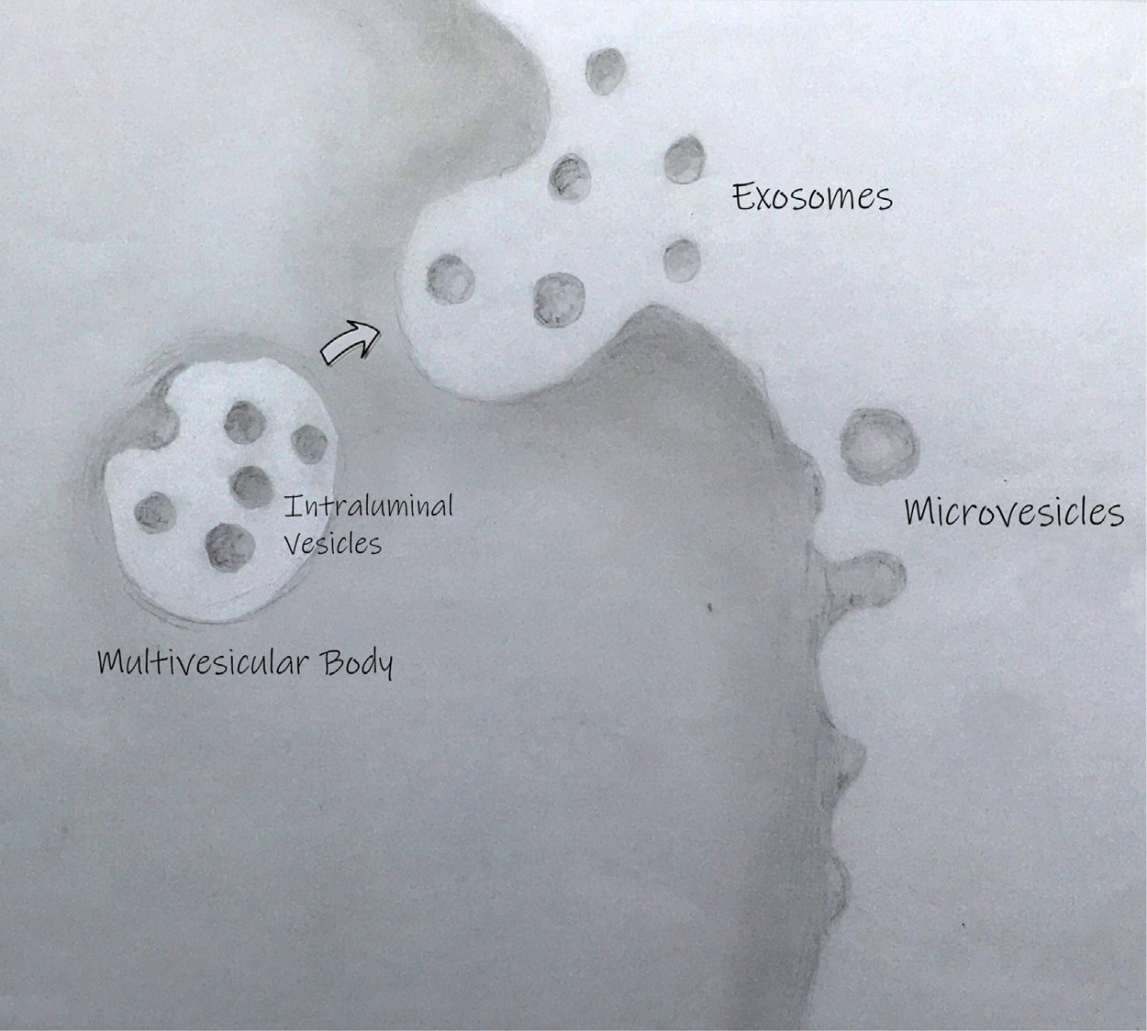
The primary routes of extracellular vesicle biogenesis. Exosomes are released from cells when a multivesicular body (which is formed when an early endosome matures and inwardly buds to form intraluminal vesicles) fuses with the plasma membrane. Ectosomes (more commonly called microvesicles and microparticles) are formed when the plasma membrane buds outwardly and pinches off. Cargo can be loaded into both intraluminal vesicles (which are released as exosomes) and ectosomes. Other types of vesicles such as apoptotic bodies (not shown) can be released by dying cells.

Between the mid‐1960s and early 1980s other early electron microscopy studies described structures consistent with the sub‐micron size of EVs. In the summer of 1966, Sun described vesicle‐like structures released from alveolar cells into the alveolar space (Sun, 1966). In the late 1960s, H. Clarke Anderson and Ermanno Bonucci described ‘matrix vesicles’. These small membrane‐bound vesicles of different sizes are embedded in the matrix of hypertrophic cartilage and could potentially play a role in bone mineralisation (Anderson, 1969; Bonucci, 1967). Nunez et al., and Gershon (1974) described the presence of small (1‐10 nm) extracellular vesicles in the bat thyroid gland during arousal from hibernation (Nunez et al., 1974). In fact, this paper was one of the first to describe the presence of multivesicular bodies (MVBs) close to the apical membrane. The authors proposed that “*fusion of the outer or limiting membrane of the multivesicular body with the apical plasma membrane might lead to the release of the vesicles contained within the structure into the luminal space*” (Nunez et al., 1974). Indeed, we now define a subtype of EV, commonly called the exosome or small EV, as being formed when the endosomal MVB structure fuses with the plasma membrane, leading to the release of the intraluminal vesicles (for an illustration of the different types of EVs *see* Figure 1).

In addition to these experiments where vesicles were found in a happenstance manner, others were specifically looking for vesicles. Between 1950 and 1970 there were several researchers who were hoping to prove that viruses caused diseases beyond infection, specifically that they caused cancer. In looking for ‘virus‐like particles’ in biofluids they often came across particulate matter (Levine et al., 1967; Seman et al., 1971) but could not identify anything they thought might actually be viral in nature (Dmochowski et al., 1968; Haguenau, 1959; Levine et al., 1967). Moreover, the particles seemed to be present in control fluids as well as those from cancer patients (Fawcett, 1956; Lunger et al., 1964; Prince & Adams, 1966). By the mid‐1960s the consensus was that it was unlikely that particles found in biofluids were attributable to viruses but were rather an artefact of separation (Prince & Adams, 1966). Finally, in 1975 Dalton published a paper studying fractions of filtered and unfiltered foetal bovine serum and demonstrated that the sera held similar particles to an epithelial cell line. He put an end to the reign of the virus‐like particle by saying that ‘*to call structures with the morphology of normally occurring vesicles of multivesicular bodies and of microvesicles associated with epithelial cells “virus‐like” is unwarranted*’ (Dalton, 1975).

Studies in other organisms suggested that vesicular structures extruded from cells were not unique to mammals. A study of *Ochromonas danica*, a flagellated alga, revealed the presence of a range of vesicles that could be visualised budding from cells and isolated by centrifugation (Aaronson et al., 1971). Preparations including EVs released by the yeast *Candida tropicalis* were shown to decrease growth of other cultures of yeast (Chigaleichik et al., 1977). Different kinds of vesicles were shown to be released by Corynebacterium, some of which were shown to induce cell agglutination (Vysotskii et al., 1977); Acinetobacter, which were seen to release phospholipid‐rich EVs (Käppeli & Finnerty, 1979); and the gram negative bacteria, *Escherichia coli*, which was shown to produce EVs containing lipopolysaccharide complexes (Käppeli & Finnerty, 1979). Whilst these studies began to unravel the ultrastructure of cells and the potential existence of EVs the research had yet to gain the momentum to unite as a cohesive field.

## 2 THE START OF SOMETHING BIG SMALL

The early 1980s mark the start of the era of expansion and more specific understanding in EV research. Whilst the significant explosion of papers, theories, arguments about nomenclature and EV‐related societies wouldn't begin for another 20 years or so, the cohesion began here. Two seminal and complementary papers published by the Johnstone and Stahl laboratories made a watertight case for the release of intraluminal vesicles from the cell, and defined them as exosomes (Harding et al., 1983; Pan & Johnstone, 1983). Whilst these papers are now considered seminal and the origins of our field, Rose herself felt the discovery to be happenstance, saying they had an ‘*Alice in Blunderland approach which led to the discovery of exosomes*’ (Johnstone, 2005). Both laboratories were using reticulocyte maturation as a model; Stahl's group to investigate membrane trafficking, and Johnstone's lab to study the biochemistry of the plasma membrane. Their work showed that during reticulocyte maturation the transferrin receptor was lost via the release of vesicles. Cliff Harding, then an MD/PhD in the Stahl laboratory, produced some stunning EM images demonstrating that these vesicles were released from the lumen of MVBs upon fusion with the plasma membrane. Conceptually, the Harding et al. (1983) paper revealed the existence of a novel intracellular sorting and trafficking pathway, now referred to as the exosome secretion pathway. Although Trams et al., and Heine (1981) originally coined the term ‘exosome’ to describe EVs shed from the surface of the cell (Trams et al., 1981), Rose Johnstone applied the name to those vesicles specifically released following fusion of MVBs with the plasma membrane and in this context the name caught on (Johnstone et al., 1987; Witwer & Théry, 2019).

As well as defining one of the hallmarks of EV vernacular, an early lecture by Rose Johnstone may have been responsible for the global opinion of EVs as just ‘waste disposal mechanisms’ for the ensuing decade. In 1991 she gave the Jeanne Manery‐Fisher Memorial Lecture which she titled *‘Maturation of reticulocytes: formation of exosomes as a mechanism for shedding membrane proteins’* which was primarily based on her paper from the same year where she suggested that exosomes were a ‘*major route for externalization of obsolete membrane proteins*’ (Johnstone et al., 1991). This paper demonstrated the presence of the transferrin receptor on exosomes, and the presence of the nucleoside transporter. The authors demonstrated that different cellular stresses resulted in the internalization and shedding of these membrane components at different times. Whilst they did not speculate on the mechanisms of this, the message that this was a way for the cells to shed ‘obsolete’ proteins stuck in the minds of researchers for some years to come.

Despite this, these early studies laid the foundation for the explosion of interest that followed over the next 35 years. In terms of the period between these seminal papers and the start of the massive expansion in EV research at the millennium, form seemed to come before function. Articles on platelet derived microparticles, microvesicles and exosomes dominated, with some important early advances in the understanding of the fundamental nature of EVs. These early studies demonstrated lateral diffusion of lipids and proteins in vesicle membranes (Gawrisch et al., 1986) and the presence and function of flippases (Vidal et al., 1989). Studies revealed glimpses of the iconic components of EVs we know today such as Rab, ARF (Vidal & Stahl, 1993) and the tetraspanins (Escola et al., 1998). As early as 1986 there were concerns about storage of blood and its effects on the EV population (George et al., 1986). In addition to work on mammalian EVs, a wealth of knowledge was developed about bacterial EVs in studies from Liverpool on *Porphyromonas gingivalis* (Kay et al., 1990; Smalley & Birss, 1987; Smalley et al., 1988, 1989). These last papers demonstrated not only the presence of bacterial EVs but the interaction of these EVs with mammalian cells in the body (Kay et al., 1990).

During the 1980s and 1990s several articles reported the quantification of EVs, demonstrating altered EV numbers in disease. The phenomenon started around 1993 with a paper on elevated microparticles in transient brain ischemia and other infarctions (Lee et al., 1993), but goes on to be explored in diseases such as angina (Singh et al., 1995) and Crohn's (Powell et al., 1996). Papers describing the physical and biochemical characteristics of EVs also began to emerge. Rose Johnstone's 1989 paper demonstrated exosomes released from reticulocytes are enzymatically active (Johnstone et al., 1989). Membrane vesiculation was shown to be a potentially protective mechanism to prevent cell lysis (Iida et al., 1991), and a way of specifically exposing phosphatidyl serine to enhance clotting (Chang et al., 1993). It was also revealed that other active enzymes could exist in EVs (Fourcade et al., 1995). Outside the field of platelet biology, it was discovered that EVs from immune cells are capable of presenting antigen (Raposo et al., 1996). This last paper, in particular, was a watershed moment that caught the imagination of many and helped to catalyse increased interest in the field of EVs. It showed that EVs had the potential to be harnessed as anti‐tumoral vaccines; indeed, this study led the Amigorena lab to investigate whether dendritic cells secrete EVs that, when loaded with tumor peptides, can eradicate tumours (Zitvogel et al., 1998), and led to clinical trials over the next decade (Escudier et al., 2005). Importantly, it showed that EVs could play functional roles in biological processes. Taken together, these ideas that EVs could have physiological roles, that they could be used as biomarkers, and that they could have therapeutic applications, led to the explosion of interest in EVs in the early 21st century.

## 3 A ROSE BY ANY OTHER NAME

In 2018 Roy and colleagues performed a systematic survey of all the papers published in the field since 2000, demonstrating the exponential growth of the field since the millennium (Roy et al., 2018). This included not only thousands of papers but also patent applications and grant funding. The specific search criteria to isolate key papers for this current review identified 1017 articles published in the 15 years between 1985 and 2000, and more than four times that number in the 10 years to 2010. The issue, still plaguing the field today, although vastly improving (Witwer & Théry, 2019), was the issue of nomenclature (Box 1) (Gould & Raposo, 2013; Witwer & Théry, 2019). Of the > 4000 papers from 2000 to 2010 the most popular search term was ‘microparticles’. This proves challenging as a search criterium because not only can it refer to platelet microparticles, but also microparticles of iron oxide (frequently used as an imaging agent) and synthetic microparticles for drug delivery. Sifting out the relevant papers remained challenging. During this period ‘exosomes’ remained more popular than ‘microvesicles’ or ‘ectosomes’ (respectively 945, 664 and 261 papers; though it should also be noted that the term ‘exosome’ also describes RNA‐processing machinery). The term ‘extracellular vesicles’ was barely seen at all with a mere 31 papers.

**BOX 1 – EV NOMENCLATURE**

In the early years of the field, a variety of terms were used to describe the structures that were observed, including ‘extracellular microvesicles’, ‘microparticles, ‘pequenas particulas’ (small particles), and ‘virus‐like particles’. The term ‘exosome’ was first used in the context of EVs by Trams *et al* (Trams et al., 1981) to describe vesicles that are produced directly by outward budding at the plasma membrane. Later, Rose Johnstone used the term ‘exosome’ to describe vesicles released following the fusion of MVBs with the plasma membrane (Johnstone et al., 1987), and this has become ISEV's recommended term for this type of vesicle (Théry et al., 2018). As the field grew, and understanding of the variety of biogenesis pathways increased, it became clear that distinct and precise nomenclature was required (Gould & Raposo, 2013). It was suggested that the catch‐all term ‘extracellular vesicles’ should be used to describe non‐replicating structures that are delimited by a lipid bilayer (György et al., 2011), and this was formalised into the current recommendations within the MISEV guidelines (Théry et al., 2018). Confusion in nomenclature can arise due to the assignment of arbitrary size ranges for different types of vesicle; in fact, the proposed names for different types of EVs are based on biogenesis pathways (Théry et al., 2018) (see also figure 1). The range of terms used to describe the different types of EV continues to grow, and authors should clearly define what type of EV they are referring to (Théry et al., 2018; Witwer & Théry, 2019). The issue of EV nomenclature has caused controversy over the years, and not all researchers agree with current recommendations (Witwer & Théry, 2019). The use of the term ‘exosome’ as a general term for EVs continues to pervade the literature (Roy et al., 2018), despite the fact that most (if not all) EV samples contain a heterogeneous mixture of vesicle types (Van Deun et al., 2017). This prevalence for the term ‘exosomes’ to describe EVs may be due to the anecdotally reported perception of exosomes as a more ‘desirable’ term, particularly in the context of industrial applications of EVs (Witwer & Théry, 2019). Similarly, the terms ectocytosis (Stein & Luzio, 1991), proposed to design specifically release of EVs from the plasma membrane, and ectosomes for such EVs (Cocucci & Meldolesi, 2015; Hess et al., 1999), are still less commonly used than the term ‘microvesicles’ for plasma membrane‐derived vesicles. It is therefore important that the field continues to discuss the best way to describe these exciting extracellular voyagers, and clear reporting is crucial to reduce confusion in nomenclature.

In the decade following the year 2000 the first reviews began to be published in the field of EV biology (Denzer et al., 2000; Schartz et al., 2002). The growing community of researchers started to explore the nature of EVs in more depth, investigating the proteome of EVs from various cell types (Bard et al., 2004; Théry et al., 2001; Wubbolts et al., 2003) as well as the lipidome (Subra et al., 2007). Cytokines were shown to be shed via EVs (Mackenzie et al., 2001) and EVs derived from immune cells were found to play a key role in the function of the immune system (Skokos et al., 2003; Van Niel, 2003). The increased interest in tumor‐derived EVs (Wolfers et al., 2001), combined with new knowledge of the role of EVs in the immune system, led to their potential as anti‐tumor therapy (Chaput et al., 2003). As the decade winds down, the real expansion in EV research began. Papers began to demonstrate the functional effects of EVs in vivo, protecting animal models from disease (Colino & Snapper, 2007). The functional transfer of nucleic acids was demonstrated (Ratajczak et al., 2006; Skog et al., 2008; Valadi et al., 2007), and a report that plant cells can use EVs as a means of communication was also published (An et al., 2007). The increased interest in EV‐based therapy was merged with burgeoning interest in stem cells as therapy, and 2009 saw the emergence of a plethora of papers on mesenchymal stem cell (MSC)‐derived vesicles (Bruno et al., 2009), further increasing the therapeutic opportunities afforded by EVs.

**FIGURE 2:**
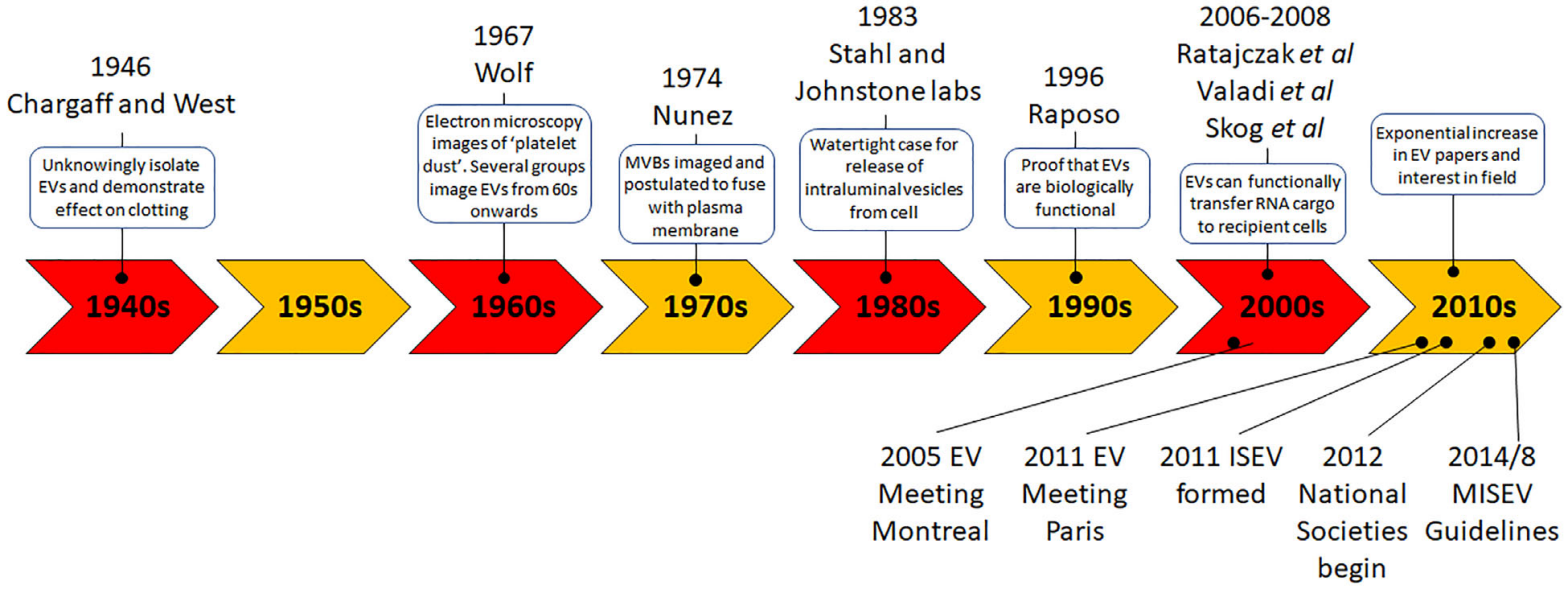
Timeline of selected milestones in the EV field

Some key EV milestones from 1940 to 2010 are summarised in Figure 2. From 2010 to today the expansion of the field has been enormous. EVs have been shown to be involved in numerous biological processes across many species, and they contribute to a plethora of diseases when deregulated. It would be unfair to pick out individual contributions to this latest decade of work as it has become so diverse and specialised, and the reader is directed to more recent reviews (Mathieu et al., 2019; Raposo & Stahl, 2019; Welsh et al., 2020).

The early 2000s also saw the first organized EV meetings take place, and the regular meetings of the International Society for Extracellular Vesicles (ISEV) now have thousands of participants working in a multitude of disciplines from all over the world. Now came the time to organize these disparate researchers and bring them together with a common purpose.

## 4 BRINGING ORDER TO THE CHAOS

The first international meeting for EVs (called exosomes at the time) was organised by Rose Johnstone and held in Montreal in 2005 (Couzin, 2005). An international meeting in 2010 in Oxford focused on advances in methodologies for measuring EVs (including new biophysical approaches such as Nanoparticle Tracking Analysis (Dragovic et al., 2011), which led to the publication of the first book on EVs (Harrison et al., 2014). A seminal moment came at a vibrant (and oversubscribed) international meeting organised by Clotilde Théry and Graça Raposo, held in Paris in 2011. At this meeting of over 200 attendees, Jan Lötvall (who fortunately was allowed to attend despite a late registration!) proposed the formation of an International Society to represent the interests of the field. Following extensive consultations with members of the community, the International Society for Extracellular Vesicles (ISEV) was formed in 2011. The first ISEV meeting was held in 2012, in Gothenburg, Sweden, and attracted more than 400 participants and was also oversubscribed. Subsequent ISEV annual meetings in Boston (2013), Rotterdam (2014, 2016), Washington (2015), Toronto (2017), Barcelona (2018) and Kyoto (2019) saw rapid growth in attendee numbers with over 1000 attendees recorded for the last 2 years. With the eruption of a global pandemic in 2020, the ISEV meeting went virtual, holding its first international online conference to great success. The society also organises and supports a variety of other focused workshops and surveys that lead to ‘position papers’ (Hill et al., 2013; Lener et al., 2015; Mateescu et al., 2017; Russell et al., 2019; Witwer et al., 2013), survey outputs (Gardiner et al., 2016; Soekmadji et al., 2018), and meeting reports (Araldi et al., 2012; Clayton et al., 2018; Hu et al., 2017; Soares et al., 2017), many of which are published in the society's ‘Journal of Extracellular Vesicles’ (JEV) (Lötvall et al., 2012). These have played an important role in helping to collate and focus the efforts of the field. This is perhaps best exemplified by the publication of ‘Minimal Information for Studies of EVs’ (MISEV) guidelines in 2014 (Lötvall et al., 2014), which has been more recently reviewed, in 2018 (Théry et al., 2018). ISEV has therefore provided an effective platform for researchers around the world to come together and share their work on EVs.

As the EV field expands, so too does the number of researchers in each country. This has led to the formation of numerous ‘National Societies’ or local networks who conduct their own local meetings and support EV research within their own countries. For a field where for many years there was considerable scepticism about whether EVs were just cellular debris, local support networks capable of validating findings and sharing new ideas, reagents, models and techniques, are crucial. These networks began in the US in 2012 with the American Society for Exosomes and Microvesicles and expanded from there; the Grupo Español de Innovación e Invesigación en Vesículas Extracelulares (in 2012), the UK, French and German Societies for EVs (in 2018), are but a few of the many national groups working together with the common goal of forwarding EV research. These National Societies help to coordinate national meetings and support regional networks of EV researchers, providing opportunities for newcomers to the field to network with established labs. Together with ISEV they provide an important support mechanism in the rich research ecosystem for the field.

ISEV has also strived to produce educational material for those new to the EV field. This includes the production of two popular and free Massive Open Online Courses (Lässer et al., 2016), the production of a 3D animated video on EV function, and posters on the basics of EVs (Nieuwland et al., 2018). This not only helps give new researchers perspective on the field, but also helps with some of the challenges and disputes the field has had, and continues to have, regarding standardization and nomenclature.

## 5 CHALLENGES

The proliferation of EV research around the world has propelled the field forwards at an ever‐increasing pace, but this brings with it a different set of problems. The EV field, as with science more generally, may suffer from a lack of reproducibility (Begley & Ellis, 2012; Neuhaus et al., 2017). This is exacerbated by the relatively young state of the field and the ‘hype’, which drives accelerated publication of ‘exciting’ new findings. The technical challenges and position papers from ISEV and other international groups have been outlined comprehensively elsewhere (Ramirez et al., 2018). Below are some of the major issues the field continues to contend with.

## 6 STANDARDISATION AND REPORTING

There is no universal agreement on many aspects of methodology in EV research, including the best methodology for enrichment, and protocols vary between laboratories (Gardiner et al., 2016). In the 1990s the International Society on Thrombosis and Haemostasis (ISTH) vascular biology subcommittee (SSC) initiated the discussion and early standardization efforts of microparticle measurements. The SSC has continued to publish important articles on pre‐analytical variables, inter‐laboratory studies and standardization of flow cytometry. On‐going standardization and collaboration between the ISTH SSC, ISAC (International society for advancement of cytometry) and ISEV continue. A recent consortium‐effort to catalogue EV research revealed a total of 1,742 experiments with 190 different isolation methods and 1,038 unique protocols to isolate EVs (Van Deun et al., 2017). While it is too early to pronounce which methodology is ‘right or wrong’, the heterogeneity in approach and frequent lack of complete reporting make comparing and interpreting the results of different studies more difficult and reaching general conclusions more challenging. This is further compounded by a lack of experimental reference materials and controls that can be reliably used to standardise experiments between labs. Initiatives such as EV‐TRACK (Van Deun et al., 2017), the MISEV guidelines (Théry et al., 2018), EV databases (Kalra et al., 2012; Simpson et al., 2012), attempts to generate reference materials (Welsh et al., 2020). ISEV taskforces and ISEV workshops on ‘Rigour and Reproducibility’ aim to address these issues, but transparency in reporting and standardisation of methodology remain two of the greatest challenges for this nascent field.

## 7 TECHNICAL CHALLENGES

There are many technical challenges associated with working on EVs which are detailed well elsewhere (Ramirez et al., 2018). Briefly, there are several techniques available for isolating EVs; they all have pros and cons, and the best choice depends on the intended downstream applications, the type of EV of interest, and level of homogeneity required (Gardiner et al., 2016). However, there is still a need to develop improved methodology to enrich higher yields, with greater homogeneity, faster time, and lower cost. The challenge is because the fluids that EVs are enriched from are typically complex matrices containing multiple contaminants, often of similar size and/or density (Ramirez et al., 2018). Improved tools are also required for characterising and quantifying EVs. A key problem here is the relatively small size of most EVs, which makes specifically counting and characterising EVs a challenge, and there is currently no perfect instrument for quantifying and characterising EVs. Another issue is their relative paucity of material when isolating EVs. To obtain sufficient material for testing using most ‘bulk methods’ (in which material from multiple vesicles is aggregated for testing), such as Western blotting, a lot of EVs are required. More sensitive methods are therefore required to make EV characterisation less onerous on laboratories. ‘Single‐EV’ methodology must be developed and improved to allow a greater range of experiments to be performed and new insights generated into EV biology. Finally, improved in vivo methods are required for studying the biology of EVs. These challenges, amongst many others, are being addressed by multiple labs around the world, and as these technical issues are addressed our ability to test hypotheses about EV function will improve.

## 8 UNANSWERED BIOLOGICAL QUESTIONS

There are some areas of EV biology where more is known, and some where almost nothing is known (Soekmadji et al., 2018). One area that needs addressing is the lack of suitable markers for specifically identifying different types of EVs. Some excellent work has been done to address this (Jeppesen et al., 2019; Kowal et al., 2016; Zhang et al., 2018). However, due in most part to the overlap in EV biogenesis mechanisms and the overlap in size and density of different EV types, it has proven difficult to generate reliable markers for different EV subtypes. Despite this, several laboratories have shown that different subtypes of EVs may exist, with different cargo, release mechanisms, and different functions (Goberdhan et al., 2019; Willms et al., 2016; Yeung et al., 2018). Better understanding of these subpopulations is a key goal for EV research over the coming decade. Another area in need of further work is EV uptake, and in particular, how EVs functionally deliver cargo to recipient cells (Mulcahy et al., 2014; Russell et al., 2019). This is thought to be a fairly low‐efficiency process, and it is understood that a significant number of EVs go to the lysosome, where they presumably are destroyed (Russell et al., 2019). The development of novel in vitro and in vivo systems for modelling EV transfer and cargo release is therefore another priority for the field. An increased understanding of cargo delivery would not only help us to understand EV biology, but it would help us to engineer vesicles specifically to avoid lysosomal destruction, resulting in the rapid emergence of strong EV therapeutic platforms.

## 9 CONCLUSIONS AND FUTURE PERSPECTIVES

Since the early electron microscopy and biochemistry studies from the 1940s through to the 1980s, the EV field has rapidly progressed. The range of functions that have been assigned to EV grows by the week. The reasons for this increased interest are manifold. The idea that these small messengers can carry cargo from one cell and deliver it for functional use by another cell is a highly attractive one that has captured the imagination. The results of many studies confirm the work of early pioneers in the field, indicating an important functional role for EVs in cell‐to‐cell communication. Their roles in many biological processes, and their deregulation in disease have fuelled further interest. EVs have been found in every biological fluid tested thus far (Carollo et al., 2019; Garcia‐Contreras et al., 2017; Jansen & Li, 2017; Lee et al., 2019; Li et al., 2019; Meng et al., 2019; O'farrell & Yang, 2019) so perhaps the greatest translational prospect for them lies in their diagnostic, prognostic and therapeutic abilities (Box 2). They have the potential to be modified for the delivery of therapeutic cargo in the treatment of different disorders (Clemmens & Lambert, 2018; Melling et al., 2019; Wiklander et al., 2019). Both their therapeutic and diagnostic potential stems from their ability to protect cargo in circulation, and their functionality as natural cell‐to‐cell transporters of multiple complex biological cargo.

**BOX 2 EVS IN DIAGNOSTICS AND THERAPEUTICS**

- The growth of the EV field has been accompanied by a growth in patents to use EVs as diagnostic markers, and therapeutic delivery vehicles. Between 2000 and 2020 there were > 500 patents filed in the US which included any of the various terms for EVs (Roy et al., 2018). As a more specific example of their use, between 2000–2020 > 30 clinical trials specified using EVs, either as diagnostic tools or as therapeutics, mainly in the field of cancer biology.
- There are two major ways that EVs might be useful as biomarkers for disease. Firstly in the acute setting as *diagnostic* markers, to determine whether someone has had an ischemic or a haemorrhagic stroke for example. And secondly in the *prognostic* setting, to help determine the course of a disease such as cancer, or the responsiveness of a patient to, for example, anti‐depressant therapy. The first CLIA/FDA approved diagnostic test using EVs is the EPI ExoDx platform, a rule‐out test for prostate cancer which uses gene expression to determine whether patients are positive for cancer‐specific markers (Mckiernan et al., 2018).
- The potential for EVs as therapeutics is vast. EVs can potentially be engineered to deliver specific therapeutics, including proteins and RNA. Non‐engineered EVs, for example those produced from MSCs, also have the potential to be used in a therapeutic context. The first clinical trials using EVs as therapeutics used autologous EVs derived from patient dendritic cells and demonstrated that EVs are capable of boosting the immune response to lung cancer in both phase I and phase II/III studies (Besse et al., 2016; Escudier et al., 2005; Morse et al., 2005). Several more trials have since been established studying the potential of several types of EVs, from autologous EVs to plant‐derived EVs in diseases from cancer to stroke (Nassar et al., 2016; Wiklander et al., 2019). While challenges remain, the potential of EVs in diagnostic and therapeutic is beginning to be unlocked and there is much excitement for the translational applications of EVs in the coming decades.

In the coming years we expect the increase in EV research observed over the past two decades to continue. This will carry on yielding incremental improvements in our knowledge of EV biology, and the translational benefits will follow.

°°

## CONFLICT OF INTEREST

Edit Buzas: Shere Gene Tharapeutics Inc. Boston, MA, US. Advisory Board Member. David Carter: Evox Therapeutics Ltd, Employee. Yong Song Gho: Founder and CEO of Rosetta Exosome, INC. Philip Stahl, Graca Raposo, Kenneth Witwer and Yvonne Couch: no conflicts of interest.
